# The HIP2~Ubiquitin Conjugate Forms a Non-Compact Monomeric Thioester during Di-Ubiquitin Synthesis

**DOI:** 10.1371/journal.pone.0120318

**Published:** 2015-03-23

**Authors:** Benjamin W. Cook, Kathryn R. Barber, Brian H. Shilton, Gary S. Shaw

**Affiliations:** Department of Biochemistry, Schulich School of Medicine & Dentistry, The University of Western Ontario, London, Ontario, Canada, N6A 5C1; George Washington University, UNITED STATES

## Abstract

Polyubiquitination is a post-translational event used to control the degradation of damaged or unwanted proteins by modifying the target protein with a chain of ubiquitin molecules. One potential mechanism for the assembly of polyubiquitin chains involves the dimerization of an E2 conjugating enzyme allowing conjugated ubiquitin molecules to be put into close proximity to assist reactivity. HIP2 (UBE2K) and Ubc1 (yeast homolog of UBE2K) are unique E2 conjugating enzymes that each contain a *C*-terminal UBA domain attached to their catalytic domains, and they have basal E3-independent polyubiquitination activity. Although the isolated enzymes are monomeric, polyubiquitin formation activity assays show that both can act as ubiquitin donors or ubiquitin acceptors when in the activated thioester conjugate suggesting dimerization of the E2-ubiquitin conjugates. Stable disulfide complexes, analytical ultracentrifugation and small angle x-ray scattering were used to show that the HIP2-Ub and Ubc1-Ub thioester complexes remain predominantly monomeric in solution. Models of the HIP2-Ub complex derived from SAXS data show the complex is not compact but instead forms an open or backbent conformation similar to UbcH5b~Ub or Ubc13~Ub where the UBA domain and covalently attached ubiquitin reside on opposite ends of the catalytic domain. Activity assays showed that full length HIP2 exhibited a five-fold increase in the formation rate of di-ubiquitin compared to a HIP2 lacking the UBA domain. This difference was not observed for Ubc1 and may be attributed to the closer proximity of the UBA domain in HIP2 to the catalytic core than for Ubc1.

## Introduction

Ubiquitin dependent proteolysis, responsible for protein degradation, utilizes ubiquitin-activating (E1), ubiquitin-conjugating (E2), and ubiquitin-ligating (E3) enzymes that sequentially activate, transfer and ligate ubiquitin (Ub) onto a lysine residue of a target protein [1]. During the middle step of this cascade ubiquitin forms a covalent thioester complex (E2~Ub) between its C-terminal carboxylate and a catalytic cysteine in the E2 enzyme. Transfer to a substrate is then mediated by either a RING E3 ligase that acts as a scaffolding protein or a HECT E3 ligase that forms a further covalent thioester complex with ubiquitin prior to substrate labeling. Repeated cycles of this process form a polyubiquitin chain using one or more of the seven available lysine residues found on ubiquitin (ie. K6, K11, K27, K29, K33, K48 and/or K63). Multiple mechanisms can give rise to polyubiquitin chain formation, utilizing different combinations of E2 and E3 enzymes [2]. For example, a sequential mechanism labels the substrate with a priming ubiquitin that is used as an anchor to add subsequent ubiquitin molecules. In some cases a separate E2 enzyme (UbcH5c = UBE2D3, Ubc4/UbcH5b = UBE2D2) is used to add the priming ubiquitin, while ubiquitin chain elongation is carried out by another E2 enzyme (CDC34 = UBE2R1, Ubc1) [3,4]. Alternatively, mechanisms have been proposed where a polyubiquitin chain is preassembled on the E2 enzyme and transferred *en bloc* to a substrate [5–7].

During ubiquitin transfer from the E2~Ub thioester intermediate it has been shown that oligomerization, either directly or assisted by an E3 enzyme, may help position two ubiquitin molecules allowing formation of an isopeptide linkage between them [6–11]. For example, the E2 enzyme Ubc13 (UBE2N) forms a heterodimer with its inactive E2 paralogue Mms2 (UBE2V2) to build K63-linked polyubiquitin chains [8–11]. Self-association of the E2 enzyme Ubc7 (UBE2G2) has been proposed to drive polyubiquitin chain formation that is likely aided by the presence of the E3 enzyme gp78 [6,7]. Two other E2 enzymes UbcH5b (UBE2D2) and UbcH5c (UBE2D3) have been shown to oligomerize in their thioester conjugate forms (UbcH5b~Ub, UbcH5c~Ub) due to interaction of the thioester bound ubiquitin of one conjugate with the “backside” of the catalytic domain of another E2 [12,13]. This oligomerization step is a requirement for polyubiquitin chain assembly [12].

The E2 enzymes HIP2 (UBE2K, human) and its yeast homolog Ubc1 function to promote ubiquitin chain extension. Elegant studies by Rodrigo-Brenni and Morgan have shown that Ubc1 functions with the anaphase-promoting complex (APC) E3 ligase to build K48-linked polyubiquitin chains on a substrate that has been initially ubiquitinated by Ubc4 [4]. This finding is consistent with earlier observations that both Ubc1 and HIP2 could synthesize unanchored polyubiquitin chains in the absence of an E3 enzyme [14,15], although this activity is markedly increased in the presence of the APC. Ubc1 and HIP2 possess unique *C*-terminal ubiquitin-associated (UBA) domains not found in other E2 enzymes. In Ubc1 the UBA domain has been shown to alter the polyubiquitin chain length [15] and in the presence of the APC the UBA domain is required for full processivity of the enzyme as shorter chains are obtained in its absence [4]. A number of experiments have shown that the UBA domains from HIP2 and Ubc1 can associate non-covalently with ubiquitin [16,17] indicating the UBA domain might be responsible for initial recognition of a pre-ubiquitinated substrate as well as facilitating subsequent ubiquitin chain building on a substrate.

One of the key intermediates in the ubiquitination process is the E2~Ub conjugate that acts as a ubiquitin donor during polyubiquitin chain building. It has been shown that the Ubc1~Ub intermediate retains the ability to recruit a ubiquitin molecule via its UBA domain [18]. Both HIP2 and Ubc1 are able to build K48-linked polyubiquitin chains in the absence of an E3 enzyme implying that the E2~Ub thioester might oligomerize to facilitate ubiquitin transfer in an analogous manner to UbcH5b~Ub and UbcH5c~Ub [12,13]. In this work, we examined the ability of the HIP2~Ub and Ubc1~Ub thioester conjugates to oligomerize in solution using a variety of biophysical techniques. Further, we show that the HIP2~Ub conjugate likely adopts an elongated structure that positions the ubiquitin and UBA domains on opposite ends from the E2 catalytic core. We demonstrate that the HIP2~Ub and Ubc1~Ub conjugates can accept ubiquitin from different sources—free untethered ubiquitin or an acceptor ubiquitin linked to an E2 enzyme. Time course experiments showed that the UBA domain in HIP2 contributes to a 5-fold enhancement in di-ubiquitin formation, while the Ubc1 UBA domain does not affect the rate of di-ubiquitin formation. Together these results provide insights into the configuration of the E2~Ub thioester conjugates for Ubc1 and HIP2 and their catalysis in the first steps of polyubiquitin chain formation.

## Experimental Procedures

### Protein Expression and Purification

Wild-type HIP2 (UBE2K, E2–25K) cDNA in a pET28a vector was obtained from the Structural Genomics Consortium (Toronto, ON). A C170S substitution in HIP2 (HIP2^C170S^) and removal of the UBA domain (HIP2Δ, residues 1–155) were introduced using the Quikchange Site-Directed Mutagenesis (Stratagene, La Jolla, CA) protocol. Ubiquitin proteins (Ub, Ub^G76C^, Ub^K48R^), Ubc1 and Ubc1Δ (residues 1–150) from *S*. *cerevisiae* were expressed and purified as previously described [15,18]. A pGEX-6P1 GST fusion vector carrying ubiquitin with an N-terminal cysteine tag (GPCLGS) was a kind gift from Dr. Leo Spyracopoulos (University of Alberta). HIP2 and HIP2Δ were expressed and purified as previously described [19]. Cysteine tagged ubiquitin proteins (GPCLGS-Ub and GPCLGS-Ub^K48R^) were expressed, purified and fluorescently labeled with Alexa-Fluor 680 C2-maleimide (Invitrogen) as previously described [20]. Disulfide complexes (HIP2–Ub^Cys^, Ubc1–Ub^Cys^) between the E2 enzymes and the C-terminal cysteine in Ub^G76C^ (Ub^Cys^) were synthesized as previously described [18]. The integrities of all proteins, Alexa-tagged proteins and E2~Ub conjugates were confirmed by electrospray ionization mass spectrometry.

### E2~Ub Conjugate Oligomerization Experiments

All protein samples were dialyzed into their respective buffers (Ubc1 and Ubc1–Ub^Cys^: 25 mM Tris-HCl, 1 mM EDTA, 150 mM NaCl at pH 7.5 (plus 2 mM TCEP for Ubc1); HIP2: 20 mM Tris-HCl, 200 mM NaCl, 1 mM TCEP at pH 8.0; HIP2–Ub^Cys^: 25 mM Tris-HCl or 100 mM NaH_2_PO_4_, 400 mM NaCl, 1 mM EDTA at pH 7.5) and diluted with dialysis buffer to the desired experimental concentrations. Sedimentation equilibrium experiments were performed using a Beckman Optima XL-A analytical ultracentrifuge equipped with an An60Ti analytical rotor with six-channel Epon-charcoal centerpieces. Experiments were performed at 5°C using rotor speeds of 15, 18, 22 and 26,000 rpm and data was analyzed using a single ideal species model [21].

Small angle X-ray scattering (SAXS) data were collected at BioCAT Beamline ID-18 of the Advanced Photon Source (Argonne, Illinois) using X-rays with a wavelength of 1.03 Å. Data were recorded using a Brandeis II or Mar165 CCD detector with a sample to detector distance of 1892 mm to 2810 mm. Two dimensional images were radially integrated using either Fit2D [22] or Igor Pro (Wavemetrics, Lake Oswego, Oregon) and further processed using Microsoft Excel or the Igor Pro macros developed at BioCAT. CRYSOL [23] was used to calculate theoretical scattering from high-resolution atomic coordinates.

### Di-ubiquitin activity assays

Reactions were performed with E1 (225 nM; Boston Biochem), Mg-ATP (10 mM), purified E2 enzymes (12 ΔM), E2–Ub disulfide complexes (10–15 μM) and Alexa-680 labeled ubiquitin or Ub^K48R^ (4–6 μM) in 50 mM Hepes buffer at pH 8.0. Alexa-labeled HIP2~Ub^K48R^ and Ubc1~ Ub^K48R^ thioester complexes were prepared using E1 (214 nM) and either Alexa-labeled Ub^K48R^ (18.3 μM) and Ubc1 (12.3 μM) or Alexa-labeled Ub^K48R^ (16.4 μM) and HIP2 (32.8 μM). Unconjugated Alexa-labeled Ub^K48R^ and E1 proteins were removed using a Sephadex G75 10/300 column (GE Biosciences). All Ubc1 thioester reactions (5 h at 37°C) contained a subset of the following: Alexa-680 dye (500 nM), ubiquitin (90 μM), Ub^K48R^ (90 μM), Ubc1–Ub^Cys^ (9.2 μM). All HIP2 thioester reactions (1 h at 37°C) contained a subset of the following final protein concentrations: Alexa-680 dye (500 nM), ubiquitin (54 μM), Ub^K48R^ (54 μM), HIP2–Ub^Cys^ (6 μM).

Time course activity assays that monitored the formation of E2~Ub^K48R^ used E1 (179 nM), HIP2 or Ubc1 (5.45 μM), HIP2Δ or Ubc1Δ (6.85 μM), Alexa-labeled Ub^K48R^ (1.6 μM), Mg-ATP (5 mM) solution and 50 mM HEPES buffer at pH 8.0, and were halted with 20 mM EDTA. HIP2-Ub_2_ complex formation was monitored by incubating Alexa-labeled HIP2~Ub^K48R^ and HIP2Δ~Ub^K48R^ with ubiquitin (16 μM), HIP2–Ub^Cys^ (16 μM) or both. This reaction scheme was repeated for Ubc1~Ub^K48R^ and Ubc1Δ~Ub^K48R^. Reaction products were separated using Bis-Tris SDS-PAGE gel electrophoresis [24] and visualized using either Coomassie blue staining or fluorescence at 700 nm using a Li-Cor Odyssey imaging system.

## Results and Discussion

**Fig 1:**
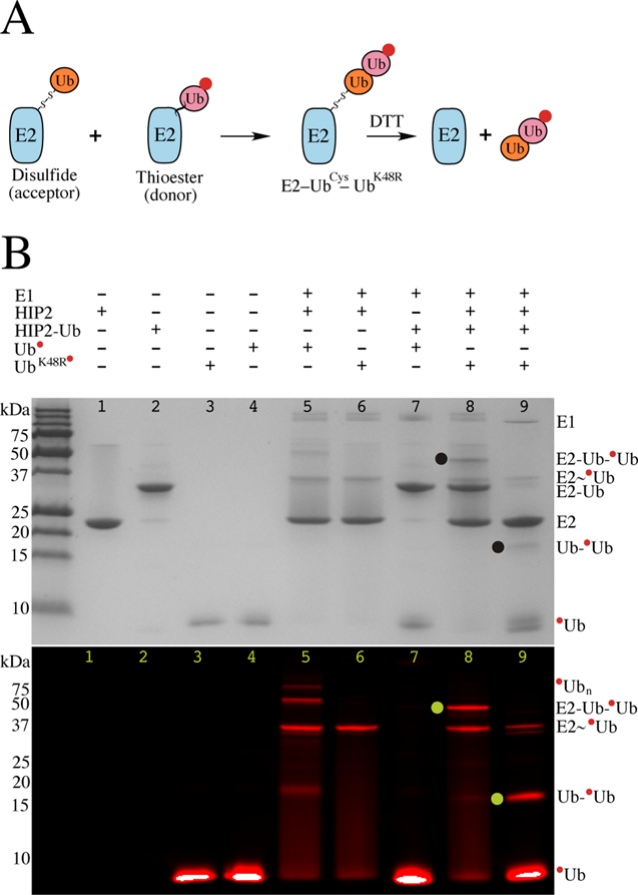
Ubiquitin transfer from the HIP2~UbK48R thioester to the HIP2–UbCys disulfide. (A) Schematic diagram showing the reaction of fluorescently labeled HIP2~Ub^K48R^ (donor, pink) with the HIP2–Ub^Cys^ disulfide (acceptor, orange) to produce HIP2–Ub^Cys^–Ub^K48R^ (HIP2–Ub_2_). In these reactions only the donor Ub^K48R^ is fluorescently labeled with Alexa-680. (B) HIP2 enzyme reactions performed with Alexa-labeled ubiquitin or Ub^K48R^ were visualized with Coomassie stain (top) or fluorescence measured at 700 nm (bottom). Reaction components are listed above each gel as described in Materials and Methods. Lane 9 shows the addition of reducing agent (10 mM DTT and 10 mM TCEP) to liberate the di-ubiquitin species. Fluorescent di-ubiquitin reaction products (E2-Ub-Ub; Ub-Ub) are indicated by the filled black / green circles. Molecular weight standards and protein species are listed to the left and right of each gel respectively.

Our experiments aimed to identify the possible role of an oligomeric E2~Ub conjugate in polyubiquitin chain formation by HIP2 (UBE2K) and its yeast homolog Ubc1. To do this we utilized stable, covalent E2-Ub disulfide complexes (E2–Ub^Cys^) to mimic the E2~Ub thioesters, acting only as a ubiquitin acceptor in a di-ubiquitin formation assay. In this manner, the E2-Ub^Cys^ molecule might also approximate a mono-ubiquitinated substrate, known to be a preferential target of both Ubc1 and HIP2 [4]. An E2~Ub conjugate carrying K48R-substituted ubiquitin (Ub^K48R^) was formed during an in situ ubiquitination reaction such that the E2~Ub^K48R^ thioester complex can only act as the ubiquitin donor. Thus the only di-ubiquitin product formation possible was an Ub^Cys^–Ub^K48R^ species conjugated to the E2 enzyme via the disulfide bond (E2–Ub^Cys^–Ub^K48R^) (Fig. 1A).

Ubiquitination assays were carried out with HIP2 and the HIP2–Ub^Cys^ covalent conjugate (Fig. 1B). Using a standard ubiquitination reaction with fluorescent labeled ubiquitin yielded the HIP2~Ub thioester as well as formation of untethered polyubiquitin species (Fig. 1B, lane 5), consistent with previous reports [25]. In the presence of fluorescent labeled Ub^K48R^, although the HIP2~Ub^K48R^ thioester was efficiently formed (lane 6), polyubiquitin species were not observed. This result is consistent with other findings that show K48-linked ubiquitin chain formation is preferential for HIP2 [14] and Ubc1 [15]. As expected, the catalytically inert HIP2–Ub^Cys^ disulfide-linked complex was unable to support polyubiquitination under similar conditions (lane 7). However, when HIP2–Ub^Cys^ was combined with fluorescent labeled HIP2~Ub^K48R^, approximately 50% of the HIP2~Ub^K48R^ was converted to HIP2–Ub_2_ (HIP2–Ub^Cys^–Ub^K48R^) (lane 8). This product did not undergo further reaction due to the K48R blockage on the distal ubiquitin. Reduction of this species yielded HIP2 and untethered Ub_2_ (Ub^Cys^–Ub^K48R^) in solution (lane 9). Similar results were observed for assays with Ubc1 and its disulfide conjugate Ubc1–Ub^Cys^ (S1 Fig.). These assays show that the transfer of ubiquitin from one E2~Ub conjugate to another E2~Ub conjugate may provide a possible route for observed di-ubiquitin chain formation by HIP2 and Ubc1, a result consistent with E2~Ub conjugate oligomerization. Alternatively, this observation could result from the E2-Ub^Cys^ complex acting simply as a mono-ubiquitinated substrate where ubiquitin is tethered by its C-terminus to the E2. These experiments also show that an E2 covalently linked to a short di-ubiquitin chain (E2-Ub_2_) can efficiently be formed, a potential advantage of a stable E2-Ub mimic that is not prone to hydrolysis like a biological E2~Ub thioester complex.

### The HIP2~Ub conjugate forms an elongated monomeric complex

**Fig 2:**
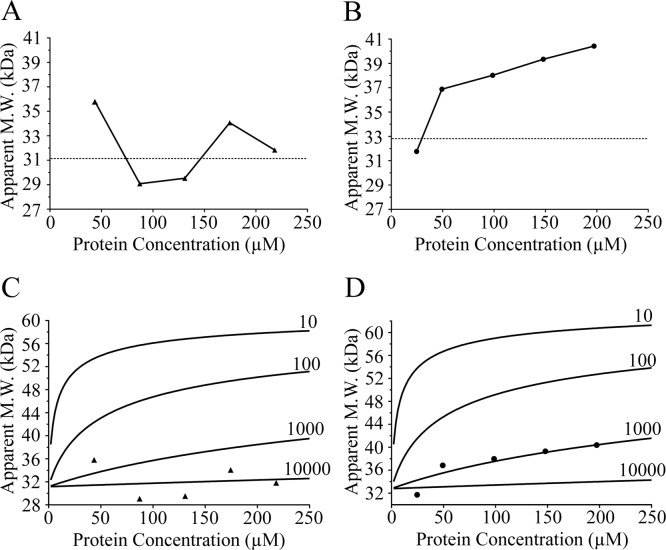
Assessment of HIP2–UbCys and Ubc1–UbCys oligomerization from SAXS data. Plots of calculated molecular weights for (A) HIP2–Ub^Cys^ (filled triangles) and (B) Ubc1–Ub^Cys^ (filled circles) as a function of protein concentration. The expected monomeric molecular weights for both E2-Ub conjugates (dotted lines) are displayed on each graph. (C, D) Calculated curves for a range of dimerization constants (*K*_d_, μM) based on apparent molecular weight versus protein concentration. The curves are superimposed over the experimental data for (C) HIP2–Ub^Cys^ and (D) Ubc1–Ub^Cys^.

The isolated E2 conjugating enzymes HIP2 and Ubc1 have been shown to be monomeric using gel filtration, sedimentation equilibrium and NMR spectroscopy [16,17,25]. Experiments to analyze the oligomerization state of the corresponding E2~Ub thioester complex can be difficult to acquire due to problems obtaining sufficient amounts of the purified E2~Ub thioester needed for biochemical characterization and the poor stabilities of some E2~Ub complexes. Previous NMR characterization of the Ubc1~Ub thioester did not indicate the presence of oligomeric states although this complex did not utilize the full length protein and hydrolyzed in only a matter of hours [26] precluding analysis by the methods described here. Different approaches to obtain more stable E2~Ub covalent complexes include converting the E2 catalytic cysteine to serine and assembling an E2~Ub ester [9], or converting the catalytic cysteine to a lysine and forming an isopeptide linkage between the E2 and ubiquitin [27] are available. The use of a disulfide bond [18] to form Ubc1–Ub^Cys^ and HIP2–Ub^Cys^ covalent complexes here allowed us to use more rigorous approaches such as sedimentation equilibrium and small angle x-ray scattering experiments (SAXS) to determine the propensity of these E2~Ub complexes to oligomerize. As shown in Table 1, sedimentation experiments for Ubc1–Ub^Cys^ and HIP2–Ub^Cys^ yielded masses within about 10% from their expected monomeric masses (S2 Fig.). Further, no obvious molecular weight trends with increasing protein concentrations, expected for an oligomerization event, were observed. As a complementary approach, SAXS experiments were used to determine the apparent masses of Ubc1–Ub^Cys^ and HIP2–Ub^Cys^ as well as the shape of the complexes. These experiments allowed us to test a broader concentration range that extended to roughly 10–20 fold higher protein concentrations than used in sedimentation equilibrium experiments. The SAXS data displayed linear Guinier plots indicating the E2-Ub conjugate species were monodisperse and free of aggregates (S3 Fig.) even at very high concentrations (up to 284 μM). Plots of apparent molecular weight as a function of protein concentration did not show any obvious oligomerization trends for HIP2–Ub^Cys^ although a small change in molecular weight (~20%) was noted for Ubc1–Ub^Cys^ (Fig. 2). To assess whether this might result from oligomerization, we calculated the expected curves for dimerization constants ranging from 10–10000 μM for both E2–Ub complexes and compared these to the data. This analysis showed that the dimerization constants for both HIP2–Ub^Cys^ and Ubc1–Ub^Cys^ were in the range of 1000 μM or greater (Fig. 2C and 2D). Together with the sedimentation equilibrium experiments these data show that the propensity for HIP2–Ub^Cys^ and Ubc1–Ub^Cys^ to oligomerize is very low in the absence of an E3 ligase protein. It is possible that oligomerization of the E2 conjugates could be modulated through recruitment with the APC although this has not been demonstrated to date.

**Table 1:** Sedimentation equilibrium results for HIP2-Ub and Ubc1-Ub covalent complexes.

E2-Ub Complex	Conc (μM)	MW_Calc_ (Da)	MW_Obs_ ^a^ (Da)
Ubc1–Ub^Cys^	14.3	32791.1	35238 ± 265
	21.5	32791.1	32536 ± 212
	43.0	32791.1	31347 ± 222
HIP2–Ub^Cys^	7.2	31163.5	34461 ± 219
	14.4	31163.5	34220 ± 169
	24.0	31163.5	34619 ± 175

^a^ Molecular weight determined using global fits of 15k, 18k, 22k and 26k rpm to a single species model.

Our data show that both HIP2–Ub^Cys^ and Ubc1–Ub^Cys^ have dimerization constants (*K*_d_ >1000 μM) that are 2–3 orders of magnitude weaker than that observed for other E2 enzymes or complexes such as Ubc13/Mms2 (2 μM) [11], UbcH5c~Ub (~25 μM) [13] and UBE2W [28] where oligomerization is considered to be important for biological function. Further, since the production of HIP2-Ub and Ubc1-Ub would be limited by the cellular concentration of ubiquitin (~10 μM) [29] and this is at least two-orders of magnitude below the dimerization constants for the E2~Ub conjugates measured here it seems unlikely that a sufficient population of HIP2-Ub and Ubc1-Ub could be formed *in vivo* that would be needed for oligomerization. These results indicate that HIP2~Ub and Ubc1~Ub polyubiquitin chain activity in our assays proceeds through a monomeric mechanism.

**Fig 3:**
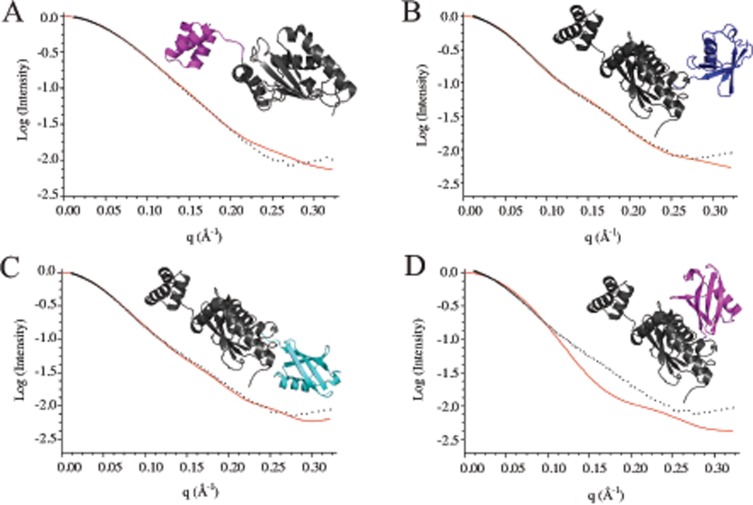
Three-dimensional models of the HIP2-Ub conjugate based on SAXS scattering data. (**A**) Scattering data points for HIP2 (142 μM, black) along with the calculated scattering curve (red) derived from the monomeric HIP2 crystal structure coordinates (PDB 1YLA). The fit was optimized by manually modifying the position of the UBA domain (magenta) with respect to the catalytic domain (grey) in HIP2. Scattering data points for HIP2-Ub^Cys^ (96 μM, black) along with the calculated scattering curves (red) determined using the relative positions of ubiquitin (blue, cyan) conjugated to HIP2 (grey) based on the coordinates for (B) UbcH5b~Ub (PDB 3JW0) and (C) Ubc13~Ub (PDB 2GMI). (D) Comparison of scattering data (black) with a more globular structure where ubiquitin is found in the closed position such as that found in a truncated form of Ubc1 (PDB 1TTE). This arrangement produced a pronounced “dip” in the calculated curve compared to experimental data. Scattering curves were calculated using the program CRYSOL.

To identify the three-dimensional arrangement of HIP2 with a conjugated ubiquitin protein we plotted our experimental SAXS scattering data and compared this to theoretical scattering curves generated from existing three-dimensional structures. Initially scattering models of the isolated HIP2 enzyme were compared with existing crystal structures [16,30]. This showed that the best fits were obtained using a monomeric model where the UBA domain had limited association with the catalytic core (Fig. 3A). Overall, this produced models that were more relaxed and elongated than observed in previously reported crystal structures. Using this model as a starting point we superimposed the E2 catalytic domain of HIP2 with the E2 domains from high-resolution models for UbcH5b~Ub (3A33), UbcH8~Ub (2KJH), UbcH5b~Ub (2JW0) and Ubc13~Ub (2GMI), while keeping the UBA domain in the same position as found in Fig. 3A. The placement of the ubiquitin molecule in each of these covalent complexes was then used to sample different positions that it might occupy in the HIP2-Ub complex. The scattering data clearly indicated that the HIP2-Ub complex does not adopt a globular conformation, denoted by a shallower curve with a “dip” at longer distances (q(Å^-1^)) (Fig. 3D). This type of structure might be expected if the UBA domain and covalently attached ubiquitin resided on the same “side” of the E2 enzyme. Instead the data was consistent with a more extended conformation where the UBA domain and conjugated ubiquitin were positioned on opposite ends of the E2 catalytic domain (Figs. 3B and 3C). The best agreement for a model of HIP2-Ub was found using the positions occupied by the ubiquitin in the Ubc13~Ub [9] and UbcH5b~Ub [31] structures (Figs. 3B and 3C). These arrangements either place the covalently attached ubiquitin in a “backbent” conformation where the ubiquitin is close to the β3- β4 loop in the E2 enzyme, as observed in Ubc13~Ub, or an “open” conformation that has little contact between the two proteins, as reported with UbcH5b~Ub. It is possible that a “closed” E2~Ub arrangement similar to that observed for a truncated version of Ubc1 [32] lacking the UBA domain would also fit the data adequately, although this would require a large re-positioning of the UBA domain from that shown in Fig. 3D. Previous work with Ubc1 shows that a conjugated ubiquitin molecule in the E2~Ub covalent complex does not interact with the UBA domain [18]. The models shown here support this finding showing the UBA domain and covalently bound ubiquitin are spaced well apart in the complex.

### HIP2 and Ubc1 have different UBA domain requirements for optimal di-ubiquitin formation

**Fig 4:**
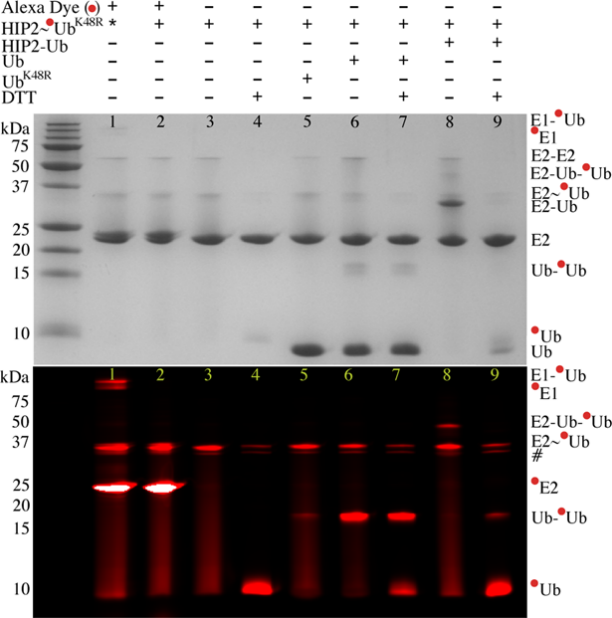
Ubiquitin and the HIP2-Ub thioester mimic act as acceptor molecules for di-ubiquitin formation. Coomassie stained (top) and fluorescent (bottom) images of Alexa-labeled HIP2~Ub^K48R^ thioester reactions. Thioester formation was performed for 30 min at 37°C using HIP2 and Alexa-labeled Ub^K48R^ as described in the Experimental Procedures. The HIP2~Ub^K48R^ thioester (lane 3) was incubated with HIP2–Ub^Cys^, ubiquitin, or Ub^K48R^ for 1 h at 37°C. Reaction components are listed above each gel with a (+) or (-). Lane 1 shows the formation of the thioester and presence of residual E1 and HIP2 as indicated by reactivity with free Alexa dye. Lane 2 shows that E1 has been removed from all subsequent reactions. Reaction products are shown to the right of the gel. Red circles denote the Alexa-labeled Ub^K48R^ molecule in each reaction. Unknown products, observed only in fluorescence-imaged gels are indicated (#).

Our physical evidence shows that in the absence of an E3 ligase enzyme Ubc1~Ub and HIP2~Ub conjugates exist primarily as monomeric complexes. In activity assays both E2~Ub conjugates were able to act as ubiquitin acceptors to build a di-ubiquitin chain tethered to the E2 enzyme (Fig. 1). To determine whether free ubiquitin might also act as an acceptor we compared the ability for ubiquitin transfer from the pre-formed HIP2~Ub (Fig. 4) and Ubc1~Ub (S4 Fig.) conjugates to either the stable E2-Ub^Cys^ disulfide or free, untethered ubiquitin. Assays where only the Ub^K48R^ moiety is fluorescently labeled in the HIP2~Ub^K48R^ thioester (Fig. 4) showed both wild-type ubiquitin and HIP2–Ub^Cys^ were able to act as acceptors to yield free Ub_2_ (Ub–Ub^K48R^, lane 6) and a K48-linked di-ubiquitin chain linked to HIP2 by a disulfide linkage (lane 8) respectively. The absence of an E1 enzyme in these reactions indicates it is not directly involved in di-ubiquitin formation as previously suggested [33].

**Fig 5:**
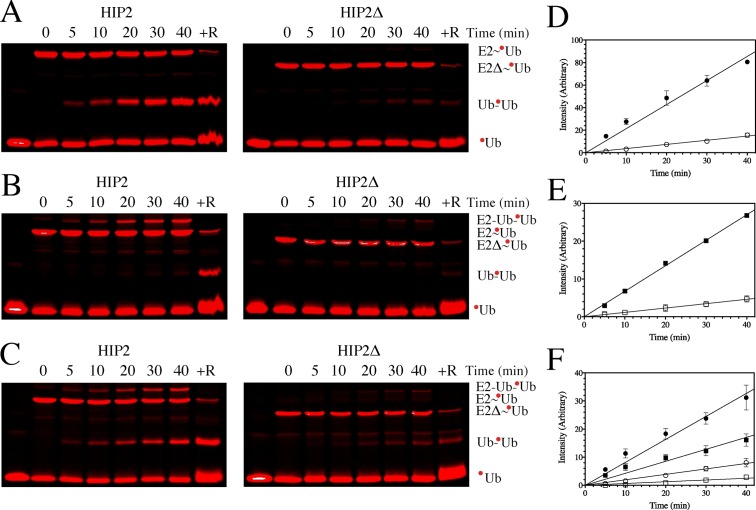
Time course reactions for HIP2~Ub thioester reactivity. Fluorescent images of SDS gels for the reaction of Alexa-labeled HIP2~Ub^K48R^ (left) or HIP2Δ~Ub^K48R^ (center) with non-fluorescently labeled (**A**) ubiquitin, (**B**) HIP2–Ub^Cys^, or (**C**) equimolar amounts of ubiquitin and HIP2–Ub^Cys^. The initial lane in each reaction shows the Alexa-labeled Ub^K48R^ alone. Fluorescently-labeled HIP2~Ub^K48R^ and HIP2Δ~Ub^K48R^ were formed through a 20 min reaction at 37°C with HIP2 or HIP2Δ and Alexa-labeled Ub^K48R^ as described in the Experimental Procedures. Thioester formation was halted with EDTA (time = 0 min) and then reacted with either ubiquitin, HIP2–Ub^Cys^ or both and samples measured at 5, 10, 20, 30 and 40 min. Reducing agent (10 mM DTT and 10 mM TCEP) was added to the 40 min sample (+R). Red circles denote the Alexa-labeled Ub^K48R^ molecule in each reaction. Also shown are the measured fluorescence intensities for (**D**) Ub_2_ formation from HIP2~Ub^K48R^ (●) or HIP2Δ~Ub^K48R^ (◯◻, (**E**) HIP2-Ub_2_ formation for HIP2~Ub^K48R^ (■) or HIP2Δ~Ub^K48R^ (◻) and (**F**) competing reactions forming Ub_2_ (●,◯) and HIP2-Ub2 (■,◻) from HIP2~Ub^K48R^ (●,■) or HIP2Δ~Ub^K48R^ (◯,◻) plotted as a function of time. Linear regression was used to fit each data set to approximate the initial product formation and the relative rates were determined by taking the ratio of the slopes. Each reaction was done in duplicate.

To determine the role of the UBA domain and to identify if there was a preference for an untethered ubiquitin or the E2–Ub^Cys^ conjugate acceptor molecule for di-ubiquitin formation, time course experiments were conducted to determine the relative reactivity of each with the HIP2~Ub^K48R^ thioester donor complexes (Fig. 5). Using the changes in fluorescence intensity, these experiments showed that the full-length HIP2~Ub^K48R^ donor complex was about five times more efficient at forming Ub-Ub^K48R^ than the E2~Ub^K48R^ donor complex lacking its UBA domain (HIP2Δ~Ub^K48R^)(Figs. 5A and 5D). Similarily, the formation of the HIP2-Ub-Ub^K48R^ species (Figs. 5B and 5E) was about five times more efficient using the full-length enzyme. This data shows that optimal formation of a di-ubiquitin species occurs when the UBA domain is present in agreement with at least one similar study [25]. Using a competition experiment, the data (Figs. 5C and 5F) also shows there is a three-fold preference for di-ubiquitin synthesis using free ubiquitin, compared with HIP2-Ub-Ub^K48R^ formation from the HIP2-Ub^Cys^ reactant, for both HIP2~Ub^K48R^ and HIP2Δ~Ub^K48R^. These experiments allow two different conclusions. The first is that free ubiquitin is a better acceptor than E2-conjugated ubiquitin. One interpretation of this is that K48 in the E2-Ub^Cys^ conjugate is partly obscured by the E2 enzyme and not available for isopeptide formation due to steric reasons. However, examination of the three-dimensional structures for UbcH5b~Ub [31] and Ubc13~Ub [9] used for our SAXS modelling, shows little evidence of this. Rather, the data in combination with our dimerization studies here suggests that association of two HIP2~Ub conjugates, a requirement to form the HIP2-Ub-Ub^K48R^ species, is not a preferred route. A second conclusion, reached many years ago by Haldeman and co-workers [25], is that the UBA domain aids but is not necessary for di-ubiquitin synthesis. Assistance by the UBA domain could be provided through non-covalent association with ubiquitin. For HIP2 and Ubc1 this has been shown to be weak [16,17] but is intensified as the polyubiquitin chain elongates [34]. Thus, the UBA domain could act as a recruitment module for a growing polyubiquitin chain on a substrate [4]. This would account for the increased efficiency for di-ubiquitin and HIP2-Ub-Ub^K48R^ formation by the full-length enzyme observed here. Our model derived from SAXS data supports these observations by placing the UBA domain opposite the catalytic site where the ubiquitin is joined in the E2~Ub thioester intermediate (Fig. 3C). In the absence of any large conformational changes in HIP2 or Ubc1 upon binding to the APC, it is conceivable that the UBA domain would be free to interact with the growing ubiquitin chain on a substrate and leave the catalytic domain unrestricted to carry-out subsequent ubiquitin additions. Conceptually, the interaction of the UBA domain with the attached ubiquitin molecules on the substrate would help initiate the formation of a processive E2~Ub/E3/Substrate-Ub^n^ complex.

The ubiquitination time courses for Ubc1 behaved differently than HIP2. For example, little difference was noted between the formation of di-ubiquitin or Ubc1-Ub-Ub^K48R^ for Ubc1 or Ubc1Δ (S5 Fig.). This supports the dispensability of the UBA domain for di-ubiquitin synthesis and parallels a previous report [29] suggesting that the Ubc1 core domain harbors the residues for recruiting and catalyzing the reaction of the acceptor ubiquitin. A possible explanation for our results is that Ubc1 has a noticeably longer linker between its catalytic and UBA domains than HIP2. It is possible that this extra length and flexibility interferes with the positioning of the acceptor ubiquitin or polyubiquitin chain and neutralizes any increase in reaction efficiency.

## Supporting Information

S1 Fig Ubc1 activity gels showing ubiquitin transfer from the Alexa-680 labeled Ubc1~UbK48R thioester onto the Ubc1-UbCys disulfide. (A) Mechanism detailing Ubc1~Ub^K48^ thioester reaction with the Ubc1-Ub^Cys^ disulfide to produce Ubc1-Ub^Cys^-Ub^K48R^ (Ubc1-Ub_2_). (B) Ubc1 enzyme reactions performed with Alexa-labeled Ub and Ub^K48R^ were visualized with Coomassie (top) or 700 nm fluorescence (bottom). Reactions were conducted as described in the text for 4 h at 37°C. Reducing agent was only added to lane 9 (10 mM DTT and 10 mM TCEP) to break the disulfide linkage. Reaction products are indicated by the filled black / yellow circles. Molecular weight standards and protein species are listed to the left and right of each gel respectively.
(PDF)

S2 Fig Sedimentation equilibrium analysis of HIP2, Ubc1, HIP2-UbCys and Ubc1- UbCys disulfide complexes. Experimental data was collected on each sample at four different rotor speeds (15k, 18k, 22k, and 26k rpm) at 5°C to determine their solution based molecular weight. Experimental data was globally fit from triplicate measurements at each rotor speed to a single species model. A representative data set (open circles) is graphed with the globally fit line (solid line) for (**A**) HIP2 (9.6 μM) at 22k rpm, (**B**) HIP2-Ub^Cys^ (24 μM) at 22k rpm, (**C**) Ubc1 (14.7 μM) at 26k rpm, and (**D**) Ubc1-Ub^Cys^ (21.5 μM) at 26k rpm. Residuals of the data points to the fit line (filled diamonds) are shown above each curve fit. The expected curve for the monomer (dotted line) and dimer (dashed line) species for HIP2-Ub^Cys^ and Ubc1-Ub^Cys^ are also plotted using fixed molecular weights and the same baseline offset as was used in that window for the global fits.
(PDF)

S3 Fig SAXS data displayed using Guinier plots. Data is presented for (A) HIP2 at 284, 213, 142, 71 and 36 μM, (B) HIP2-Ub^Cys^ at 218, 175, 131, 87 and 43 μM, (C) Ubc1 at 235, 176, 118, 58 and 29 μM and (D) Ubc1-Ub^Cys^ at 197, 148, 98, 49 and 24 μM. All data is normalized to the most concentrated data set in the series and plotted using a similar angular range (Q^2^). Each data set (open circles) was fit to the linear region to determine the forward scattering (I_0_) and radius of gyration (R_g_) reported in S1 Table.
(PDF)

S4 Fig Reaction of Alexa-680 labeled Ubc1~UbK48R with both untethered ubiquitin and Ubc1-UbCys disulfide. Coomassie stained image (top) and fluorescent image at 700 nm (bottom) of Ubc1~Ub^K48R^ thioester reactions. Thioester reactions were performed as described in the text for 30 min at 37°C and reacted with Ubc1-Ub^Cys^, ubiquitin, or Ub^K48R^. Reaction components are listed above each gel with a (+) or (-). All components for thioester formation are signified in lane 1 (*), while Ubc1~Ub^K48R^ thioester for all other lanes contain no E1 or ATP. Unconjugated E1 and E2 are visualized with reactive Alexa dye, and DTT is used for reducing disulfide bonds (lanes 4, 7 and 9). Reaction products are shown with filled black / yellow circles. Molecular weight standards are listed to the left and protein species to the right of each gel.
(PDF)

S5 Fig Time course reaction of Alexa-680 labeled Ubc1~UbK48R and Ubc1Δ~UbK48R thioester complexes with untethered ubiquitin or the Ubc1-UbCys disulfide complex. Fluorescent visualized gel (700 nm) detailing the reaction of the labeled Ubc1~Ub^K48R^ or Ubc1Δ~Ub^K48R^ with (A) ubiquitin or (B) Ubc1-Ub^Cys^. The initial lane in each reaction shows the Alexa-680 labeled Ub^K48R^ alone. Ubc1~Ub^K48R^ and Ubc1Δ~Ub^K48R^ thioester complexes were pre-formed at 37°C and halted through addition of 20 mM EDTA (t = 0 min). Time course reactions were then performed following addition of either ubiquitin (A) or Ubc1-Ub^Cys^ (B). Samples were taken at 10, 20, 40, 60 and 90 min. Reducing agent (10 mM DTT and 10 mM TCEP) was added to the 90 min sample and labeled as +R on the gel. The measured fluorescence intensities for (**C**) Ub_2_ formation from Ubc1~Ub^K48R^ (●) or Ubc1Δ~Ub^K48R^ (◯◻, and (**D**) Ubc1-Ub_2_ formation for Ubc1~Ub^K48R^ (■) or Ubc1Δ~Ub^K48R^ (◻) are plotted as a function of time. Linear regression was used to fit each data set to approximate the initial product formation and the relative rates were determined by taking the ratio of the slopes. Each reaction was done in duplicate.
(PDF)

S1 Table Small angle x-ray scattering results for Ubc1, HIP2 and their covalent ubiquitin complexes. (PDF)
