# Supplementary material for: The HIP2~Ubiquitin Conjugate Forms a Non-Compact Monomeric Thioester during Di-Ubiquitin Synthesis
Source: PLoS One. 2015 Mar 23;10(3):e0120318. doi: 10.1371/journal.pone.0120318 (PMC4370575; doi:10.1371/journal.pone.0120318)
Supplement: S1 Fig — (A) Mechanism detailing Ubc1~UbK48 thioester reaction with the Ubc1-UbCys disulfide to produce Ubc1-UbCys-UbK48R (Ubc1-Ub2). (B) Ubc1 enzyme reactions performed with Alexa-labeled Ub and UbK48R were visualized with Coomassie (top) or 700 nm fluorescence (bottom). Reactions were conducted as described in the text for 4 h at 37°C. Reducing agent was only added to lane 9 (10 mM DTT and 10 mM TCEP) to break the disulfide linkage. Reaction products are indicated by the filled black / yellow circles. Molecular weight standards and protein species are listed to the left and right of each gel respectively. (PDF) [file pone.0120318.s001.pdf]

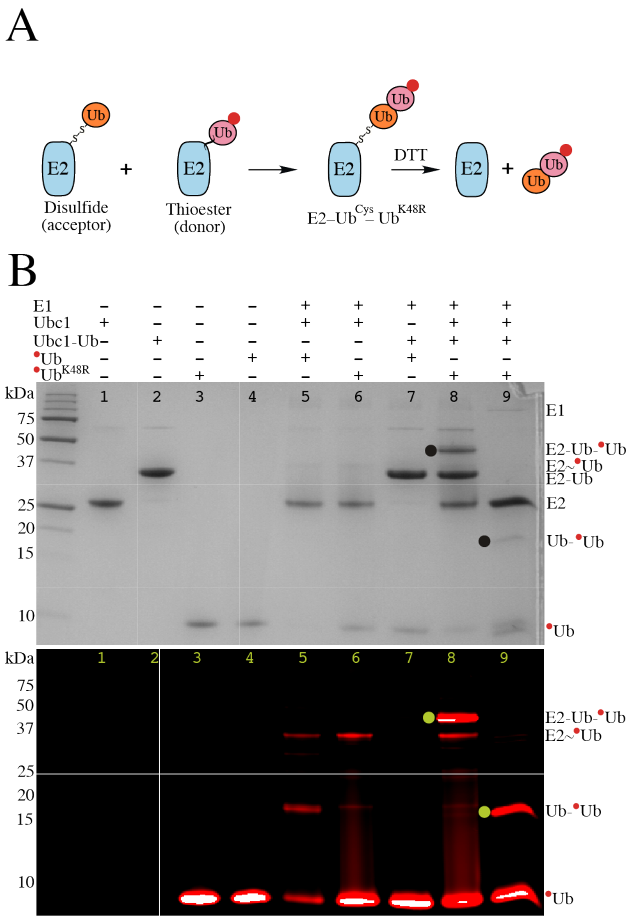

Figure S1. Ubc1 activity gels showing ubiquitin transfer from the Alexa-680 labeled Ubc1~Ub^{K48R} thioester onto the Ubc1-Ub^{Cys} disulfide. (A) Mechanism detailing Ubc1~Ub^{K48} thioester reaction with the Ubc1-Ub^{Cys} disulfide to produce Ubc1-Ub^{Cys}-Ub^{K48R} (Ubc1-Ub2). (B) Ubc1 enzyme reactions performed with Alexa-labeled Ub and Ub^{K48R} were visualized with Coomassie (top) or 700 nm fluorescence (bottom). Reactions were conducted as described in the text for 4 h at 37 °C. Reducing agent was only added to lane 9 (10 mM DTT and 10 mM TCEP) to break the disulfide linkage. Reaction products are indicated by the filled black / yellow circles. Molecular weight standards and protein species are listed to the left and right of each gel respectively.
