# Supplementary material for: The HIP2~Ubiquitin Conjugate Forms a Non-Compact Monomeric Thioester during Di-Ubiquitin Synthesis
Source: PLoS One. 2015 Mar 23;10(3):e0120318. doi: 10.1371/journal.pone.0120318 (PMC4370575; doi:10.1371/journal.pone.0120318)
Supplement: S2 Fig — Experimental data was collected on each sample at four different rotor speeds (15k, 18k, 22k, and 26k rpm) at 5°C to determine their solution based molecular weight. Experimental data was globally fit from triplicate measurements at each rotor speed to a single species model. A representative data set (open circles) is graphed with the globally fit line (solid line) for (A) HIP2 (9.6 μM) at 22k rpm, (B) HIP2-UbCys (24 μM) at 22k rpm, (C) Ubc1 (14.7 μM) at 26k rpm, and (D) Ubc1-UbCys (21.5 μM) at 26k rpm. Residuals of the data points to the fit line (filled diamonds) are shown above each curve fit. The expected curve for the monomer (dotted line) and dimer (dashed line) species for HIP2-UbCys and Ubc1-UbCys are also plotted using fixed molecular weights and the same baseline offset as was used in that window for the global fits. (PDF) [file pone.0120318.s002.pdf]

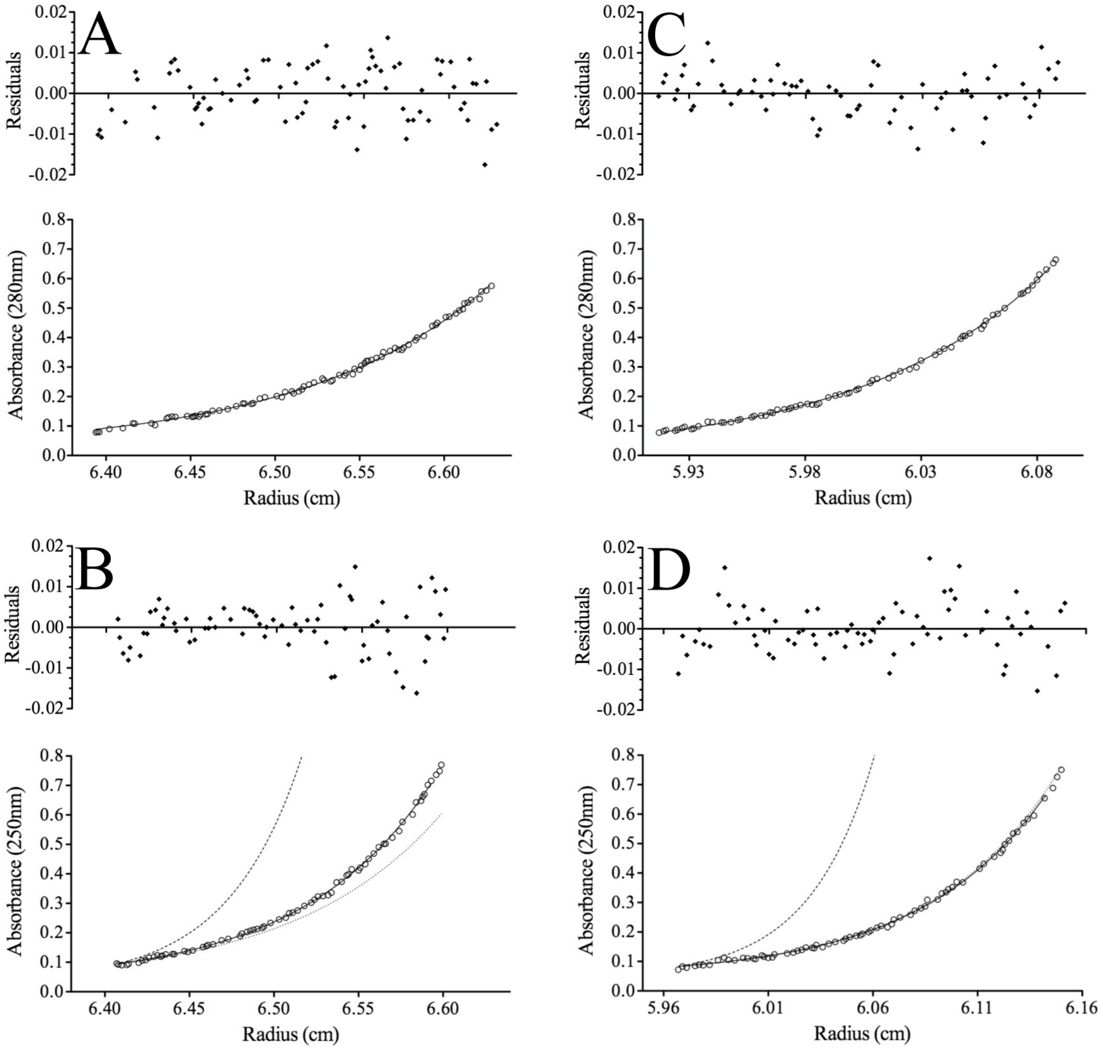

Figure S2. Sedimentation equilibrium analysis of HIP2, Ubc1, HIP2-Ub^{Cys} and Ubc1-Ub^{Cys} disulfide complexes. Experimental data was collected on each sample at four different rotor speeds (15k, 18k, 22k, and 26k rpm) at 5 °C to determine their solution based molecular weight. Experimental data was globally fit from triplicate measurements at each rotor speed to a single species model. A representative data set (open circles) is graphed with the globally fit line (solid line) for (**A**) HIP2 (9.6 μM) at 22k rpm, (**B**) HIP2-Ub^{Cys} (24 μM) at 22k rpm, (**C**) Ubc1 (14.7 μM) at 26k rpm, and (**D**) Ubc1-Ub^{Cys} (21.5 μM) at 26k rpm. Residuals of the data points to the fit line (filled diamonds) are shown above each curve fit. The expected curve for the monomer (dotted line) and dimer (dashed line) species for HIP2-Ub^{Cys} and Ubc1-Ub^{Cys} are also plotted using fixed molecular weights and the same baseline offset as was used in that window for the global fits.
