# Supplementary material for: The HIP2~Ubiquitin Conjugate Forms a Non-Compact Monomeric Thioester during Di-Ubiquitin Synthesis
Source: PLoS One. 2015 Mar 23;10(3):e0120318. doi: 10.1371/journal.pone.0120318 (PMC4370575; doi:10.1371/journal.pone.0120318)
Supplement: S3 Fig — Data is presented for (A) HIP2 at 284, 213, 142, 71 and 36 μM, (B) HIP2-UbCys at 218, 175, 131, 87 and 43 μM, (C) Ubc1 at 235, 176, 118, 58 and 29 μM and (D) Ubc1-UbCys at 197, 148, 98, 49 and 24 μM. All data is normalized to the most concentrated data set in the series and plotted using a similar angular range (Q2). Each data set (open circles) was fit to the linear region to determine the forward scattering (I0) and radius of gyration (Rg) reported in S1 Table. (PDF) [file pone.0120318.s003.pdf]

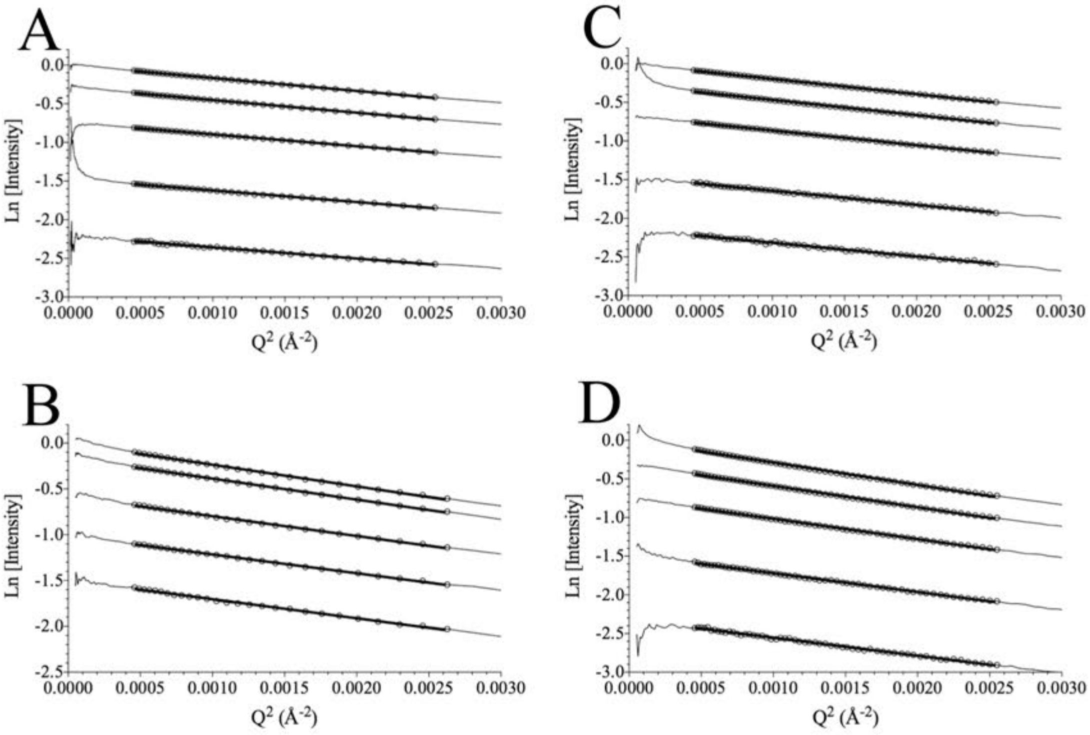

Figure S3. SAXS data displayed using Guinier plots. Data is presented for (A) HIP2 at 284, 213, 142, 71 and 36 μM, (B) HIP2-Ub^{Cys} at 218, 175, 131, 87 and 43 μM, (C) Ubc1 at 235, 176, 118, 58 and 29 μM and (D) Ubc1-Ub^{Cys} at 197, 148, 98, 49 and 24 μM. All data is normalized to the most concentrated data set in the series and plotted using a similar angular range (Q^2). Each data set (open circles) was fit to over the linear region to determine the forward scattering (I_0) and radius of gyration (R_g) reported in Table S1.
