# Supplementary material for: The HIP2~Ubiquitin Conjugate Forms a Non-Compact Monomeric Thioester during Di-Ubiquitin Synthesis
Source: PLoS One. 2015 Mar 23;10(3):e0120318. doi: 10.1371/journal.pone.0120318 (PMC4370575; doi:10.1371/journal.pone.0120318)
Supplement: S4 Fig — Coomassie stained image (top) and fluorescent image at 700 nm (bottom) of Ubc1~UbK48R thioester reactions. Thioester reactions were performed as described in the text for 30 min at 37°C and reacted with Ubc1-UbCys, ubiquitin, or UbK48R. Reaction components are listed above each gel with a (+) or (-). All components for thioester formation are signified in lane 1 (*), while Ubc1~UbK48R thioester for all other lanes contain no E1 or ATP. Unconjugated E1 and E2 are visualized with reactive Alexa dye, and DTT is used for reducing disulfide bonds (lanes 4, 7 and 9). Reaction products are shown with filled black / yellow circles. Molecular weight standards are listed to the left and protein species to the right of each gel. (PDF) [file pone.0120318.s004.pdf]

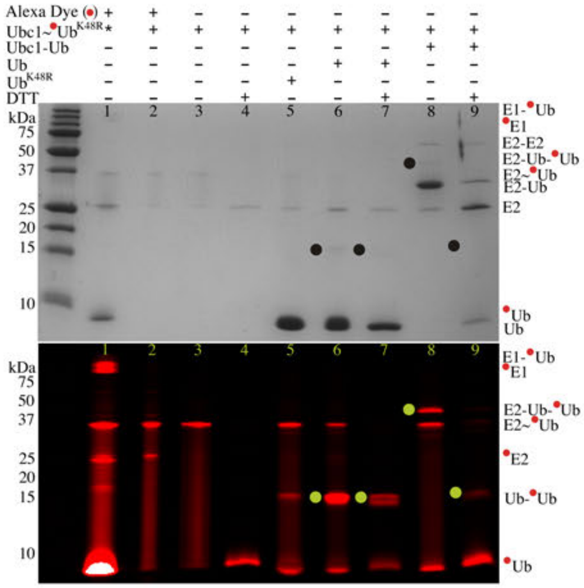


**Figure S4**. **Reaction of Alexa-680 labeled Ubc1~Ub$^{K48R}$ with both untethered ubiquitin and Ubc1-Ub$^{Cys}$ disulfide.** Coomassie stained image (top) and fluorescent image at 700 nm (bottom) of Ubc1~Ub$^{K48R}$ thioester reactions. Thioester reactions were performed as described in the text for 30 min at 37 °C and reacted with Ubc1-Ub$^{Cys}$, ubiquitin, or Ub$^{K48R}$. Reaction components are listed above each gel with a (+) or (-). All components for thioester formation are signified in lane 1 (*), while Ubc1~Ub$^{K48R}$ thioester for all other lanes contain no E1 or ATP. Unconjugated E1 and E2 are visualized with reactive Alexa dye, and DTT is used for reducing disulfide bonds (lanes 4, 7 and 9). Reaction products are shown with filled black / yellow circles. Molecular weight standards are listed to the left and protein species to the right of each gel.
