# Supplementary material for: The HIP2~Ubiquitin Conjugate Forms a Non-Compact Monomeric Thioester during Di-Ubiquitin Synthesis
Source: PLoS One. 2015 Mar 23;10(3):e0120318. doi: 10.1371/journal.pone.0120318 (PMC4370575; doi:10.1371/journal.pone.0120318)
Supplement: S5 Fig — Fluorescent visualized gel (700 nm) detailing the reaction of the labeled Ubc1~UbK48R or Ubc1Δ~UbK48R with (A) ubiquitin or (B) Ubc1-UbCys. The initial lane in each reaction shows the Alexa-680 labeled UbK48R alone. Ubc1~UbK48R and Ubc1Δ~UbK48R thioester complexes were pre-formed at 37°C and halted through addition of 20 mM EDTA (t = 0 min). Time course reactions were then performed following addition of either ubiquitin (A) or Ubc1-UbCys (B). Samples were taken at 10, 20, 40, 60 and 90 min. Reducing agent (10 mM DTT and 10 mM TCEP) was added to the 90 min sample and labeled as +R on the gel. The measured fluorescence intensities for (C) Ub2 formation from Ubc1~UbK48R (●) or Ubc1Δ~UbK48R (◯◻, and (D) Ubc1-Ub2 formation for Ubc1~UbK48R (■) or Ubc1Δ~UbK48R (◻) are plotted as a function of time. Linear regression was used to fit each data set to approximate the initial product formation and the relative rates were determined by taking the ratio of the slopes. Each reaction was done in duplicate. (PDF) [file pone.0120318.s005.pdf]

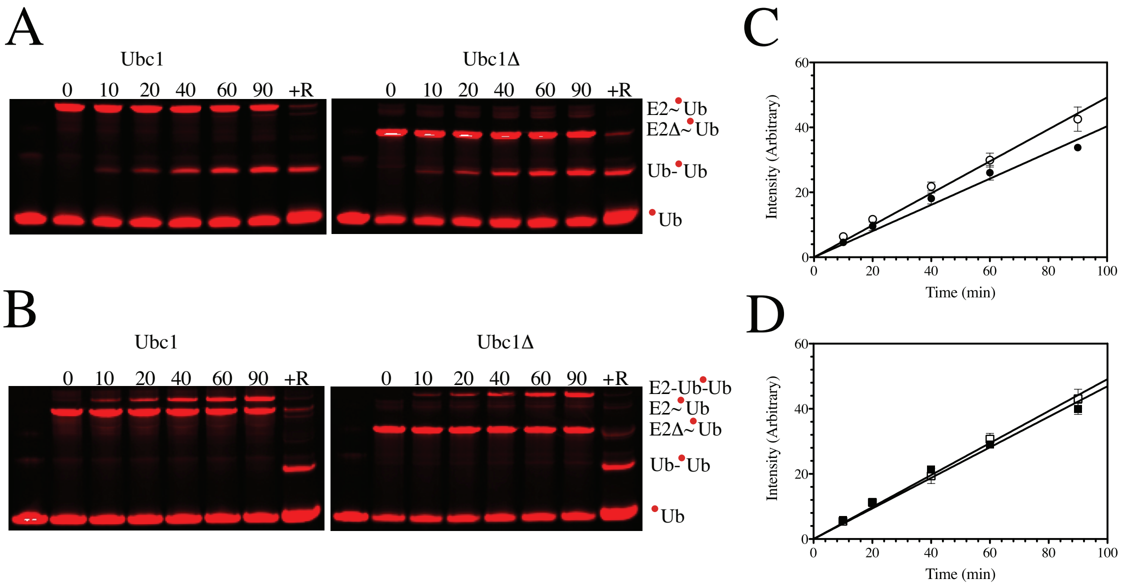

Figure S5. Time course reaction of Alexa-680 labeled Ubc1~Ub^{K48R} and Ubc1Δ~Ub^{K48R} thioester complexes with untethered ubiquitin or the Ubc1-Ub^{Cys} disulfide complex. Fluorescent visualized gel (700 nm) detailing the reaction of the labeled Ubc1~Ub^{K48R} or Ubc1Δ~Ub^{K48R} with (A) ubiquitin or (B) Ubc1-Ub^{Cys}. The initial lane in each reaction shows the Alexa-680 labeled Ub^{K48R} alone. Ubc1~Ub^{K48R} and Ubc1Δ~Ub^{K48R} thioester complexes were pre-formed at 37 °C and halted through addition of 20 mM EDTA (t=0 min). Time course reactions were then performed following addition of either ubiquitin (A) or Ubc1-Ub^{Cys} (B). Samples were taken at 10, 20, 40, 60 and 90 min. Reducing agent (10 mM DTT and 10 mM TCEP) is added to the 90 min sample and labeled as +R on the gel. The measured fluorescence intensities for (**C**) Ub_2 formation from Ubc1~Ub^{K48R} (●) or Ubc1Δ~Ub^{K48R} (○), and (**D**) Ubc1-Ub_2 formation for Ubc1~Ub^{K48R} (■) or Ubc1Δ~Ub^{K48R} (□) are plotted as a function of time. Linear regression was used to fit each data set to approximate the initial product formation and the relative rates were determined by taking the ratio of the slopes. Each reaction was done in duplicate.
