# Supplementary material for: The HIP2~Ubiquitin Conjugate Forms a Non-Compact Monomeric Thioester during Di-Ubiquitin Synthesis
Source: PLoS One. 2015 Mar 23;10(3):e0120318. doi: 10.1371/journal.pone.0120318 (PMC4370575; doi:10.1371/journal.pone.0120318)
Supplement: S1 Table — (PDF) [file pone.0120318.s006.pdf]

Table S1. Small angle x-ray scattering results for Ubc1, HIP2 and their covalent ubiquitin complexes

Protein	Conc. (μM)	R_g (A)	MW_{Obs}[a,b] (kDa)	MW_{Exp} (kDa)
Ubc1	29	23.1	28.3	24.2
	58	23.4	27.8	24.2
	118	23.8	30.5	24.2
	176	24.3	30.5	24.2
	235	24.3	30.0	24.2
Ubc1-Ub^{Cys}	24	26.5	31.8	32.8
	49	27.0	36.9	32.8
	98	28.1	38.0	32.8
	148	28.8	39.3	32.8
	197	29.5	40.4	32.8
HIP2	36	20.8 ± 0.8	24.3 ± 0.4	22.5
	71	21.2 ± 0.4	25.5 ± 0.2	22.5
	142	21.4 ± 0.2	26.4 ± 0.1	22.5
	213	22.2 ± 0.1	27.8 ± 0.1	22.5
	284	22.4 ± 0.1	27.8 ± 0.1	22.5
HIP2-Ub^{Cys}	43	24.7 ± 0.2	35.8 ± 0.2	31.2
	87	24.5 ± 0.1	29.1 ± 0.1	31.2
	131	24.9 ± 0.1	29.5 ± 0.1	31.2
	175	26.0 ± 0.1	34.1 ± 0.1	31.2
	218	26.3 ± 0.1	31.8 ± 0.1	31.2

[a]Molecular weight determined by comparison to cytochrome C standard samples

[b]Error analysis for data analyzed by Igor Pro (HIP2 and HIP2-Ub^{Cys}). Error analysis based on I_0 errors.
